# Computer vision syndrome-related symptoms in presbyopic computer workers

**DOI:** 10.1007/s10792-023-02724-z

**Published:** 2023-04-27

**Authors:** Caridad Galindo-Romero, Celia L. Rodríguez-Zamora, Diego García-Ayuso, Johnny Di Pierdomenico, Francisco J. Valiente-Soriano

**Affiliations:** 1 Departamento de Oftalmología, Facultad de Medicina, Universidad de Murcia and Instituto Murciano de Investigación Biosanitaria Pascual Parrilla (IMIB-Pascual Parrilla), Murcia, Spain; 2 Facultad de Óptica y Optometría, Universidad de Murcia, Murcia, Spain; 3 Grupo de Oftalmología Experimental, Instituto Murciano de Investigación Biosanitaria-Pascual Parrilla, Edificio LAIB Planta 5ª, Carretera Buenavista s/n, El Palmar, 30120 Murcia, Spain

**Keywords:** Computer vision syndrome, Presbyopia, Ergonomic factors, Electronic devices

## Abstract

**Purpose:**

To evaluate the prevalence of computer vision syndrome (CVS)-related symptoms in a presbyopic population using the computer as the main work tool, as well as the relationship of CVS with the electronic device use habits and the ergonomic factors.

**Methods:**

A sample of 198 presbyopic participants (aged 45–65 years) who regularly work with a computer completed a customised questionnaire divided into: general demographics, optical correction commonly used and for work, habits of electronic devices use, ergonomic conditions during the working hours and CVS-related symptoms during work performance. A total of 10 CVS-related symptoms were questioned indicating the severity with which they occurred (0–4) and the median total symptom score (MTSS) was calculated as the sum of the symptoms.

**Results:**

The MTSS in this presbyopic population is 7 ± 5 symptoms. The most common symptoms reported by participants are dry eyes, tired eyes and difficulties in refocusing. MTSS is higher in women (*p* < 0.05), in laptop computer users (*p* < 0.05) and in teleworkers compared to office workers (*p* < 0.05). Regarding ergonomic conditions, MTSS is higher in participants who do not take breaks while working (*p* < 0.05), who have an inadequately lighting in the workspace (*p* < 0.05) and in the participants reporting neck (*p* < 0.01) or back pain (*p* < 0.001).

**Conclusion:**

There is a relationship between CVS-related symptoms, the use of electronic devices and the ergonomic factors, which indicates the importance of adapting workplaces, especially for home-based teleworkers, and following basic visual ergonomics rules.

**Supplementary Information:**

The online version contains supplementary material available at 10.1007/s10792-023-02724-z.

## Introduction

Computer vision syndrome (CVS), also called digital eye strain, is a set of visual and ocular symptoms that emerges in subjects after prolonged use of electronic devices (ED), including computers, laptops, tablets, e-readers or smartphones [1–3], and results in a worsening quality of life [3, 4]. The most important CVS-related symptoms are: as ocular or external symptoms, mostly related to dry eye disease, irritation, tearing, burning or dryness [2, 3, 5, 6]; and as visual or internal symptoms, mostly related to refractive, accommodative or convergence disorders, eye strain, blur, diplopia, headache or sensitivity to light [1, 3, 4]. Also, CVS is frequently associated with musculoskeletal symptoms, such as neck, back or shoulder pain [4, 7], or sleep disruption due to the higher exposure to blue light [8, 9].

CVS has recently been studied in several populations, including university students [10–16] and office workers [11, 14, 17–21]. The most frequent symptoms related to CVS are dry eye and eyestrain [19], and it has also been correlated with daily hours of ED usage [19, 22], the type of optical correction used [23] and, in case of computer users, with ergonomic conditions such as position, tilt and glare [22, 24, 25].

Among all office workers, presbyopic subjects have become the focus of CVS study due to: (1) age and gender, greater in older women [1, 22, 26], mainly due to dry eye disease [21, 27, 28]; (2) the optical correction needed for far, intermediate and near vision, in most cases corrected with progressive spectacles [17, 18, 25, 29, 30]; and (3) the relationship between ergonomic conditions and the type of optical correction for ED usage, mainly the computer position while wearing progressive spectacles [18, 22, 25].

During the last years, there has been an increase in the use of ED worldwide, especially the smartphone, since all ED are used for both leisure and work tasks [31]. Since the lockdown due to COVID-19 was decreed in Spain in March 2020 [32], this increase has accelerated because most people had to work or study remotely from home [14, 33]. As a consequence of this increase, recent studies have described a worsening of CVS-related symptoms in many countries [14, 15, 33–36]. A previous study carried out in Spain in subjects of all ages described that CVS-related symptoms in the Spanish population were greater in participants who worked remotely from home, spent more daily hours using ED and spent less time outdoors [14]. Furthermore, this work showed that older participants reported a higher prevalence of CVS-related symptoms [14] and many people in Spain have remained working remotely from home after the strict lockdown due to COVID-19.

Therefore, the aim of this study is to analyse in depth the prevalence of CVS-related symptoms in these presbyopic population using a computer screen as their main work tool and its association with other ergonomic variables such as work habits, like the workplace or the type and habits of ED use.

## Methods

### Participants

To collect the symptoms reported by presbyopic participants, a web-based questionnaire was designed and hosted by Google Forms (Google Inc., CA, USA) and disseminated through the social networks of the University of Murcia via a link. To achieve greater dissemination of the questionnaire among the target population, employees from the University of Murcia (teaching, research or administrative staff) were encouraged to fill in the questionnaire and to resend it to their contacts once they had completed it.

The research was approved by the Ethics Committee of the University of Murcia and carried out in accordance with the principles of the Declaration of Helsinki.

The inclusion criteria were: presbyopic participants (age between 45 and 65 years old) who used a computer as their main work tool (both desktop and laptop) and were willing to participate. A total of 259 responses were collected from participants with no identifiable data of which 61 were excluded for analysis because they did not work with a computer although they were included in the age range.

### Questionnaire

The questionnaire was available during the months of February and March 2021 (2 months). The questionnaire could be completed in less than 5 min, included a total of 20 questions and was organised in different sections (see Appendix 1 in electronic supplementary material): general demographic information (age, sex and main working tool), general data on the optical correction commonly used and utilised for working, usage habits of ED, ergonomic conditions during the working hours and CVS-related symptoms reported by the participants during the performance of their work following the questionnaire developed by Hayes et al. [3]. Participants were asked to rate a total of 10 CVS-related symptoms indicating the severity with which they occurred: ‘never’ scored 0, ‘sometimes’ 1 point, ‘half the time’ 2 points, ‘most of the time’ 3 points and ‘always’ 4 points. Therefore, the maximum score that each person could obtain was 40 points. Based on previous studies [3, 19], scores of 8 or higher were considered as CVS. All data were then exported to Excel spreadsheets for the analysis (Microsoft Excel, Microsoft Corporation, Redmond, WA, USA).

### Statistical analysis

Data were analysed using GNU PSPP software (version 1.4.1. Free Software Foundation. Boston, MA). Mean ± standard deviation (SD) is shown for normally distributed variables, such as age. Median total symptom score (MTSS) ± interquartile range (IQR) is shown for non-normally distributed total symptom score. The nonparametric Kruskal–Wallis test was used for quantitative variables with two or more groups. Multivariate testing was performed for gender, place of work and total daily hours of ED use. The strength of association for significant factors is summarised using *β* values and *p* values. In all cases, the level of significance was set at *p* value < 0.05.

## Results

The demographic characteristics are shown in Table 1. Of the 198 participants, 125 were female (63.1%) and 73 males (36.9%), with a median ± IQR of 54 ± 8 years.

**Table 1:** Demographic characteristics and median total symptom score (MTSS) ± interquartile range (IQR) of the sample (*N* = 198)

	*N*	%	MTSS	IQR	*p* value
Gender					
Female	125	63.1	8	5	< 0.05*
Male	73	36.9	6	5	
Age					
45–49	45	22.7	8	6	0.36
50–54	59	29.8	7	6	
55–59	58	29.3	7	5.25	
> 60	36	18.2	6.5	4.75	
Optical correction					
Spectacles	158	79.8	7	6	0.34
Contact lens	2	1.0	6	6	
Both	17	8.6	7	7	
None	21	10.6	7.5	5.5	
Optical correction for computer work					
Single near-vision lenses	69	34.8	7.5	7	0.64
Bifocal lenses	6	3.0	10	10	
Progressive lenses	67	33.8	6	4	
Occupational lenses	16	8.1	5	4.75	
None (had surgery)	2	1.0	5	4.75	
None at near vision (myopia at far distance)	19	9.6	8	7	
Contact lenses	19	9.6	7	5	
Total	198	100			

*p* value obtained from Kruskal–Wallis test, * when *p* < 0.05

The MTSS of all participants was 7 ± 5, ranged from 0 to 40 symptoms; according to Hayes et al. [3] the results showed that MTSS was significantly higher in females than in males (8 ± 5 vs 6 ± 5, respectively, *p* < 0.05). In particular, ‘dry eyes’ (*p* < 0.01), ‘headache’ (*p* < 0.05) and ‘tired eyes’ (*p* < 0.01) were significantly higher in females. No significant differences were observed between ages (*p* = 0.36) (see Table 1).

Participants were asked to indicate the type of optical correction they usually used in their daily routine. No significant differences in MTSS were found among participants who used only spectacles (*N* = 158, 79.8%), contact lenses (*N* = 2, 1%), both spectacles and contact lenses (*N* = 17, 8.6%) or no optical correction (*N* = 21, 10.6%).

For computer work, most participants used single near-vision lenses (*N* = 69, 34.8%) or progressive lenses (*N* = 67, 33.8%). Although there were no significant differences between the different types of optical correction, it was observed that participants using bifocal lenses (*N* = 6, 3%) or single near-vision lenses had a higher MTSS (10 ± 10 and 7.5 ± 7 symptoms, respectively) than those using progressive or occupational lenses (6 ± 4 and 5 ± 4.75 symptoms, respectively) (see Table 1).

### Computer vision syndrome-related symptoms

The percentage of participants that reported each symptom and its severity is summarised in Table 2. All participants reported at least one symptom and an average of 62.6% reported to experience symptoms ‘half of the time’ or more frequent, in particular, tired eyes (17.2%), dry eyes (16.7%), difficult to refocusing (12.6%) or blurred distance vision after computer work (11.1%).

**Table 2:** Prevalence (%) and severity of computer vision syndrome-related symptoms of the sample (*N* = 198)

	None of the time	Some of the time	Half of the time	Most of the time	All of the time
Blurred vision while viewing the computer	0.0	100	0.0	0.0	0.0
Blurred distance vision after computer work	39.9	45.5	11.1	2.5	1.0
Difficulty or slowness in refocusing from one distance to another	26.3	54.5	12.6	4.0	2.5
Irritated or burning eyes	43.9	42.4	9.6	4.0	0.0
Dry eyes	41.9	35.4	16.7	4.0	2.0
Eyestrain	68.2	24.7	5.1	2.0	0.0
Headache	42.4	50.5	4.0	2.0	1.0
Tired eyes	18.2	59.1	17.2	5.1	0.5
Sensitivity to bright lights	51.5	33.3	8.6	4.0	2.5
Eye discomfort	41.4	47.5	7.6	3.0	0.5

### Effect of working conditions and electronic device use

Of all participants, the MTSS was significantly lower in office workers (*N* = 125, 63.1%, 7 ± 4.5 symptoms) than in-home teleworkers (*N* = 73, 36.9%, 8 ± 5 symptoms, *p* < 0.05). In particular, the symptom ‘difficulty or slowness in refocusing from one distance to another’ was significantly higher in-home teleworkers (*p* < 0.05) (see Table 3).

**Table 3:** Working habits, electronic device usage and median total symptom score (MTSS) ± interquartile range (IQR) of the sample (*N* = 198)

	*N*	%	MTSS	IQR	*p* value
Place of working					
Office	125	63.1	7	4.5	< 0.05*
Home	73	36.9	8	5	
Type of computer					
Desktop	79	39.9	6	4	< 0.05*
Laptop	62	31.3	9	6	
Both	57	28.8	8	6	
Daily hours of computer use for working					
< 6 h	81	40.9	6	7	0.21
≥ 6 h	117	59.1	8	4	
Daily hours of electronic device use					
0–5 h	43	21.7	6	6	0.21
6–10 h	119	60.1	7	6	
> 10 h	36	18.2	8	6.25	
Perceived worsening of symptoms while teleworking					
Yes	41	20.7	9	6.5	< 0.001***
No	66	33.3	6	6	
Always teleworking	18	9.1	10	5.25	
Always office working	73	36.9	7	4	
Total	198	100			

*p* value obtained from Kruskal–Wallis test, *p < 0.05 and ***  < 0.001

Moreover, the MTSS was significantly higher when the laptop was used (*N* = 62, 31.3%, 9 ± 6 symptoms) compared to the use of desktop computer (*N* = 79, 39.9%, 6 ± 4 symptoms) or the use of both computers (*N* = 57, 28.8%, 8 ± 6 symptoms) (*p* < 0.05). In particular, ‘dry eyes’ (*p* < 0.01) and ‘tired eyes’ (*p* < 0.05) were significantly higher in laptop users (see Table 3).

Regarding the daily hours of computer use for work, there was a tendency of a higher MTSS for participants who worked more than 6 h/day with the computer (*N* = 117, 59.1%, 8 ± 4 symptoms) than in participants who worked less than 6 h/day (*N* = 81, 40.9%, 6 ± 7 symptoms). However, no significant differences were observed (*p* = 0.21) (see Table 3).

A similar result was observed for the total daily hours of ED use. Of all participants, those who used ED for 0–5 h/day (*N* = 43, 21.7%) had a MTSS of 6 ± 6 symptoms, lower than participants who used ED for 6–10 h/day (*N* = 119, 60.1%, 7 ± 6 symptoms) or more than 10 h/day (*N* = 36, 18.2%, 8 ± 6.25 symptoms). These differences did not reach statistical significance (*p* = 0.21) (see Table 3). However, ‘tired eyes’ was significantly higher in participants who used ED more than 10 h/day (*p* < 0.001).

In addition, home teleworkers were asked if they had noticed any change in their CVS-related symptoms compared to when they worked in their office. Participants who were always home teleworkers (*N* = 125, 63.1%) had a significantly higher MTSS (8 ± 5 symptoms) than those who has previously worked at office (*N* = 73, 36.9%, 7 ± 4 symptoms, *p* < 0.001). Likewise, participants who reported a worsening of symptoms (*N* = 41, 20.7%) had a significantly higher MTSS (9 ± 6.5, respectively) than those who did not perceived a worsening of symptoms (*N* = 66, 33.3%, 6 ± 6 symptoms) (see Table 3).

### Multivariate analysis for gender, place of work and total daily hours of electronic device use

Multiple linear regression analysis revealed that MTSS was significantly correlated with gender (standardised *β* = 0.145; *p* < 0.05) and total daily hours of ED use (standardised *β* = 0.139; *p* < 0.05) but no significant correlation was found for MTSS with place of work (standardised *β* = 0.120; *p* = 0.09). Therefore, female gender and higher daily time of ED use were found to cause a greater predisposition to CVS-related symptoms.

### Effect of the ergonomic conditions

Participants were asked to report on the ergonomic conditions at work (see Table 4). Of all the participants, those who reported to have an adequate lighting (*N* = 181, 91.4%, 7 ± 5 symptoms) had a significant lower MTSS than participants who did not have adequate lighting (*N* = 10, 5.1%, 10 ± 6 symptoms, *p* < 0.05). A similar result was observed in terms of having an adequate workspace, although in this case no significant differences were found (*p* = 0.22) (see Table 4).

**Table 4:** Ergonomic conditions and median total symptom score (MTSS) ± interquartile range (IQR) of the sample (*N* = 198)

	*N*	%	MTSS	IQR	*p* value
Adequate lighting					
Yes	181	91.4	7	5	< 0.05*
No	10	5.1	10	6	
Don’t know	7	3.5	9	8	
Adequate workspace					
Yes	175		7	5	0.22
No	11		9	6	
Don’t know	12		6.5	5	
Frequency of breaks while working					
30 min	29	14.6	6	4	< 0.01**
1 h	47	23.7	7	4	
More than 1 h	86	43.4	8	6.25	
No breaks	36	18.2	8	7	
Screen position in relation to eye level					
At eye level	120	60.6	7	4	0.42
Above the eye level	11	5.6	9	7	
Below the eye level	67	33.8	7	6.5	
Neck pain					
Yes	84	42.4	8	5.75	< 0.01**
No	106	53.5	6	5	
No data	8	4			
Back pain					
Yes	97	49.0	9	6	< 0.001***
No	93	47.0	6	3.5	
No data	8	4			
Total	198	100			

*p* value obtained from Kruskal–Wallis test, *p < 0.05, ** *p* < 0.01 and *** *p* < 0.001

There was also a relationship between the MTSS and the frequency of breaks while working with the computer, which was higher in participants who took breaks less frequently. Between participants who did not take breaks (*N* = 36, 18.2%, 8 ± 7 symptoms) and those who took breaks every 30 min (*N* = 29, 14.6%, 6 ± 4 symptoms) there was a statistically significant difference (*p* < 0.01). In particular, ‘headache’ was significantly lower in this last group (*p* < 0.01). Regarding the computer use while working, there was a trend of increased symptoms in participants who had the screen above the eye level (*N* = 11, 5.6%, 9 ± 7 symptoms) compared to those who had the screen at the eye level (*N* = 120, 60.6%, 7 ± 4 symptoms) or below the eye level (*N* = 67, 33.8%, 7 ± 6.5 symptoms), although no significant differences were observed (*p* = 0.42).

Finally, participants were asked to report whether they complained of neck or back pain after work. There was a significant relationship between participants who had neck (*p* < 0.01) or back pain (*p* < 0.001) and higher MTSS (see Table 4). Moreover, the following symptoms were significantly higher in participants who had neck or back pain: ‘dry eyes’ (*p* < 0.01), ‘tired eyes’ (*p* < 0.01), ‘headache’ (*p* < 0.01) and ‘sensitivity to bright lights’ (*p* < 0.01).

## Discussion

This study demonstrates that the main risk factors to develop CVS in presbyopic subjects using the computer as their main work tool are gender, significantly greater in women; the workspace, greater in-home-based teleworkers and inadequate or deficiently illuminated workspaces; the type and usage habits of ED, greater in laptop users and subjects who spend more daily hours using ED; and not taking breaks while working.

CVS has become the focus of the study over the past few years due to the massive use of ED for all activities and therefore, the increased prevalence of CVS among the entire population [4, 37].

In this study, a total of 198 presbyopic subjects filled in the survey. In order to compare the severity of CVS, the MTSS was calculated according to Hayes et al. [3], considering CVS when MTSS is equal to or higher than 8. Our results indicate a mean MTSS of 7 ± 4.5 symptoms. Among the studied population, severe CVS is not observed, but the importance of these results lies in the risk factors that are associated with it and make the MTSS higher, for instance, gender. In agreement with previous studies, our results show that MTSS is significantly higher in women (8 ± 5 symptoms) than in men (6 ± 5, *p* < 0.05) [1, 10, 19, 22, 26, 38]. Although there is a small imbalance in the proportion of female and male participants, the statistic is robust and consistent. This result can be explained by two factors: the higher prevalence of dry eye disease described in older women [21, 27, 28] and the worsening of dry eye symptomatology related to the use of ED recently described [39, 40].

Between age ranges, no statistical differences were found, but there is a tendency for younger presbyopic participants to report more symptoms, according to a previous study with early presbyopic subjects ED users that are more symptomatic, highlighting the need for early correction to avoid CVS [18].

One of the controversies about CVS and presbyopic patients is the type of optical correction used for computer work [37]. The results of this study indicate a trend of higher CVS-related symptoms in those participants who use bifocal (10 ± 10 symptoms) or single near-vision lenses (7.5 ± 5 symptoms), than those who use progressive or occupational lenses who have lower CVS-related symptoms (6 ± 4 symptoms), but none of these comparisons reach statistical significance. Previous studies described the beneficial effect of multifocal lenses for computer use compared to monofocal lenses [17, 18], although a recent systematic review did not describe any significant evidence in this regard and further research is needed to elucidate the best optical correction [37]. Previous studies have also described that CVS is significantly higher in subjects who wear contact lenses compared to those who wear spectacles [1, 28]. In this study, out of 198 participants, only 2 used contact lenses and their MTSS is 7 ± 5 (see Table 1). Therefore, there is no data enough to demonstrate the worsening of CVS in contact lenses users.

Previous studies have also described a relationship between CVS and the daily hours of ED use, and the type of ED used [14, 19, 22, 38]. In this study, no significant differences are shown in terms of daily hours of computer use for working or total daily hours of ED usage, although a slight tendency for users with higher exposure to show more symptoms, reaching a MTSS of 8 ± 6.25 in the subjects who used the computer more than 10 h/day. However, it can be observed that laptop computer users report significantly higher CVS symptomatology (9 ± 6) than desktop computer users (6 ± 4, *p* < 0.05).

Moreover, the lockdown due to the COVID-19 pandemic has exacerbated the increase of ED use, at the same time has encouraged teleworking from home [14, 34, 40]. In this study, 54% of the participants started teleworking from home since the lockdown and showed a significantly higher MTSS than office workers (8 ± 5 vs 7 ± 4.5, respectively). Moreover, home-based teleworkers reported a perceived worsening of CVS-related symptoms since they began teleworking, similar to the previous study that described a perceived worsening of symptoms across the age population, that may be related to the reduced time participants spent outdoors [14, 35].

Another factor contributing to increased CVS in-home-based teleworkers could be poor ergonomic conditions in terms of lighting. In this study, the results are in accordance with Sanchez-Brau et al. [25] that observed that poor workplace lighting (measured with a luxmeter) was associated with a higher rate of subjects with CVS symptomatology [22].

Similar results have been reported in university students taking online classes and showing a worsening of CVS-related symptoms [15, 34] as well as an increase of dry eye [36, 39, 40].

One of the important issues affecting presbyopes and CVS are musculoskeletal symptoms that manifest themselves due to optical correction, posture and angulation relative to the screen. In this study, participants who report back or neck pain are more likely to have a higher incidence of CVS-related symptoms, similar to the results of other studies [41]. One of the reasons that could explain this relationship is the position and angulation of the monitor in combination with the type of optical correction used for working, as described [22]. Previous studies have also described that participants whose eye level is above the screen while working have a lower incidence of CVS-related symptoms [10, 16]. However, in this study, no significant changes are observed but MTSS is slightly higher when the screen is above the eye level [41].

Clinical management of CVS remains one of the most studied issues at present [4]. During the past few years, several interventions are being investigated to treat CVS, such as the type of optical correction, the use of blue-blocking filters, artificial tears or dietary supplements [4, 37]. However, the efficacy of these interventions is not conclusive [42]. To date, ergonomic interventions have proven to be the most effective [4], therefore, the identification of the ergonomic variables that most affect the development of CVS is essential to advance the understanding of CVS, as this study has shown that workspace, computer type and lighting influence CVS. Therefore, these results show conditions or risk factors for CVS-related symptoms that are common for presbyopic workers who use the computer as their main work tool and that can be reduced through appropriate prevention. This fact is exportable and general for all presbyopic computer workers all over the world, not only in Spain since conditions and workplaces are comparable among developed countries.

In addition to the specifications on the ED usage to comply with safe use recommendations [43], one of the ergonomic rules recommended by eye care practitioners to prevent CVS-related symptoms is to take breaks during the ED use [2]. In this study, the results confirm that CVS-related symptoms are significantly lower when participants take breaks more frequently [10, 14, 16]. Considering these data, it is important to highlight preventive measures to avoid or reduce the incidence of CVS in the population.

Finally, this study has some limitations: (1) Sample size used in this study is limited. This fact may explain the absence of a significant trend towards an increase in CVS-related symptoms in relation to young presbyopes [18] or the daily time of ED use [14, 19] described by other authors. In addition, the final number of participants showed a slight gender imbalance, with 63% of the participants being female and 37% male. (2) The results were taken from a survey, therefore there is no data of the previous visual health of the participants, i.e. whether participants already had any visual disfunction that could contribute to CVS or whether participants have any ocular pathologies, such as cataracts, dry eye syndrome or macular degeneration which may influence the participant’s complaints. In addition, one of the biases of this study could be that the subjects who responded the survey are more likely to suffer from CVS-related symptoms, which would overestimate the prevalence. Moreover, the lack of clinical measurements prevents us from knowing the state of optical correction wore by the participants. It would be interesting for further studies to analyse the worsening of CVS-related symptoms taking these variables into account, such as visual disfunction, ocular pathologies or optical correction type. (3) No measurements of the ergonomic conditions in the workspace were carried out. However, previous studies have described that CVS is affected by the workplace lighting or ergonomic postures [22] consistent with the results of this study.

In conclusion, this study demonstrates which ergonomic factors worsen CVS-related symptoms in presbyopic subjects who use the computer as their main work tool such as home teleworkers who have poor workstation conditions, being inadequate or poorly lit. These working conditions should be reviewed and improved to prevent the development of these symptoms. One of the main missions of eye practitioners should be to educate and make the population aware of the need to take care of their visual health through visual and ergonomic standards that improve CVS-related symptomatology.

## Supplementary Information

Below is the link to the electronic supplementary material.

Supplementary file1 (DOCX 35 KB)

## References

[1] Blehm C, Vishnu S, Khattak A, Mitra S, Yee RW (2005). Computer vision syndrome: a review. Surv Ophthalmol.

[2] Sheedy J, Shaw-McMinn PG (2003). Diagnosing and treating computer-related vision problems.

[3] Hayes JR, Sheedy JE, Stelmack JA, Heaney CA (2007). Computer use, symptoms, and quality of life. Optom Vis Sci.

[4] Coles-Brennan C, Sulley A, Young G (2019). Management of digital eye strain. Clin Exp Optom.

[5] Rossi GCM, Scudeller L, Bettio F, Pasinetti GM, Bianchi PE (2019). Prevalence of dry eye in video display terminal users: a cross-sectional Caucasian study in Italy. Int Ophthalmol.

[6] Nakamura S (2018). Approach to dry eye in video display terminal workers (basic science). Invest Ophthalmol Vis Sci.

[7] Helland M, Horgen G, Kvikstad TM, Garthus T, Aarås A (2011). Will musculoskeletal and visual stress change when Visual Display Unit (VDU) operators move from small offices to an ergonomically optimized office landscape?. Appl Ergon.

[8] Tosini G, Ferguson I, Tsubota K (2016). Effects of blue light on the circadian system and eye physiology. Mol Vis.

[9] Heo JY, Kim K, Fava M, Mischoulon D, Papakostas GI, Kim MJ, Kim DJ, Chang KJ, Oh Y, Yu BH, Jeon HJ (2017). Effects of smartphone use with and without blue light at night in healthy adults: a randomized, double-blind, cross-over, placebo-controlled comparison. J Psychiatr Res.

[10] Altalhi A, Khayyat W, Khojah O, Alsalmi M, Almarzouki H (2020). Computer vision syndrome among health sciences students in Saudi Arabia: prevalence and risk factors. Cureus.

[11] Canto-Sancho N, Sanchez-Brau M, Ivorra-Soler B, Segui-Crespo M (2020). Computer vision syndrome prevalence according to individual and video display terminal exposure characteristics in Spanish university students. Int J Clin Pract.

[12] Logaraj M, Madhupriya V, Hegde S (2014). Computer vision syndrome and associated factors among medical and engineering students in Chennai. Ann Med Health Sci Res.

[13] Mowatt L, Gordon C, Santosh ABR, Jones T (2018). Computer vision syndrome and ergonomic practices among undergraduate university students. Int J Clin Pract.

[14] Galindo-Romero C, Ruiz-Porras A, García-Ayuso D, Di Pierdomenico J, Sobrado-Calvo P, Valiente-Soriano FJ (2021). Computer vision syndrome in the Spanish population during the COVID-19 lockdown. Optom Vis Sci.

[15] Wang L, Wei X, Deng Y (2021). Computer vision syndrome during SARS-CoV-2 outbreak in university students: a comparison between online courses and classroom lectures. Front Public Health.

[16] Agarwal S, Goel D, Sharma A (2013). Evaluation of the factors which contribute to the ocular complaints in computer users. J Clin Diagn Res.

[17] Jaschinski W, Konig M, Mekontso TM, Ohlendorf A, Welscher M (2015). Comparison of progressive addition lenses for general purpose and for computer vision: an office field study. Clin Exp Optom.

[18] Jaschinski W, Konig M, Mekontso TM, Ohlendorf A, Welscher M (2015). Computer vision syndrome in presbyopia and beginning presbyopia: effects of spectacle lens type. Clin Exp Optom.

[19] Portello JK, Rosenfield M, Bababekova Y, Estrada JM, Leon A (2012). Computer-related visual symptoms in office workers. Ophthalmic Physiol Opt.

[20] Ranasinghe P, Wathurapatha WS, Perera YS, Lamabadusuriya DA, Kulatunga S, Jayawardana N, Katulanda P (2016). Computer vision syndrome among computer office workers in a developing country: an evaluation of prevalence and risk factors. BMC Res Notes.

[21] Sanchez-Valerio MDR, Mohamed-Noriega K, Zamora-Ginez I, Baez Duarte BG, Vallejo-Ruiz V (2020). Dry eye disease association with computer exposure time among subjects with computer vision syndrome. Clin Ophthalmol.

[22] Sanchez-Brau M, Domenech-Amigot B, Brocal-Fernandez F, Quesada-Rico JA, Segui-Crespo M (2020). Prevalence of computer vision syndrome and its relationship with ergonomic and individual factors in presbyopic VDT workers using progressive addition lenses. Int J Environ Res Public Health.

[23] Jaschinski W (2003). Asthenopic complaints and ocular convergence at the computer workstation: new test procedures for practice and research. Klin Monbl Augenheilkd.

[24] Konig M, Haensel C, Jaschinski W (2015). How to place the computer monitor: measurements of vertical zones of clear vision with presbyopic corrections. Clin Exp Optom.

[25] Sanchez-Brau M, Domenech-Amigot B, Brocal-Fernandez F, Segui-Crespo M (2021). Computer vision syndrome in presbyopic digital device workers and progressive lens design. Ophthalmic Physiol Opt.

[26] Artime-Ríos E, Suárez-Sánchez A, Sánchez-Lasheras F, Seguí-Crespo M (2022). Computer vision syndrome in healthcare workers using video display terminals: an exploration of the risk factors. J Adv Nurs.

[27] Kaido M, Kawashima M, Yokoi N, Fukui M, Ichihashi Y, Kato H, Yamatsuji M, Nishida M, Fukagawa K, Kinoshita S, Tsubota K (2015). Advanced dry eye screening for visual display terminal workers using functional visual acuity measurement: the Moriguchi study. Br J Ophthalmol.

[28] Tauste A, Ronda E, Molina MJ, Segui M (2016). Effect of contact lens use on computer vision syndrome. Ophthalmic Physiol Opt.

[29] Kolbe O, Degle S (2018). Presbyopic personal computer work: a comparison of progressive addition lenses for general purpose and personal computer work. Optom Vis Sci.

[30] Weidling P, Jaschinski W (2015). The vertical monitor position for presbyopic computer users with progressive lenses: how to reach clear vision and comfortable head posture. Ergonomics.

[31] DiTrendia DMT (2018) Informe ditrendia: Mobile en España y en el Mundo 2018. https://aso.app/wp-content/uploads/2018/09/Ditrendia-Informe-Mobile-2018.pdf

[32] BOE Real Decreto 463/2020, de 14 de marzo, por el que se declara el estado de alarma para la gestión de la situación de crisis sanitaria ocasionada por el COVID-19. https://www.boe.es/boe/dias/2020/03/14/pdfs/BOE-A-2020-3692.pdf

[33] Bahkir FA, Grandee SS (2020). Impact of the COVID-19 lockdown on digital device-related ocular health. Indian J Ophthalmol.

[34] Li R, Ying B, Qian Y, Chen D, Li X, Zhu H, Liu H (2021). Prevalence of self-reported symptoms of computer vision syndrome and associated risk factors among school students in China during the COVID-19 pandemic. Ophthalmic Epidemiol.

[35] Alabdulkader B (2021). Effect of digital device use during COVID-19 on digital eye strain. Clin Exp Optom.

[36] Mohan A, Sen P, Shah C, Jain E, Jain S (2021). Prevalence and risk factor assessment of digital eye strain among children using online e-learning during the COVID-19 pandemic: digital eye strain among kids (DESK study-1). Indian J Ophthalmol.

[37] Singh S, McGuinness MB, Anderson AJ, Downie LE (2022). Interventions for the management of computer vision syndrome: a systematic review and meta-analysis. Ophthalmology.

[38] Boadi-Kusi SB, Adueming PO, Hammond FA, Antiri EO (2022). Computer vision syndrome and its associated ergonomic factors among bank workers. Int J Occup Saf Ergon JOSE.

[39] Talens-Estarelles C, García-Marqués JV, Cervino A, García-Lázaro S (2021). Online vs in-person education: evaluating the potential influence of teaching modality on dry eye symptoms and risk factors during the COVID-19 pandemic. Eye Contact Lens.

[40] García-Ayuso D, Di Pierdomenico J, Moya-Rodríguez E, Valiente-Soriano FJ, Galindo-Romero C, Sobrado-Calvo P (2021). Assessment of dry eye symptoms among university students during the COVID-19 pandemic. Clin Exp Optom.

[41] Teo C, Giffard P, Johnston V, Treleaven J (2019). Computer vision symptoms in people with and without neck pain. Appl Ergon.

[42] Vera J, Redondo B, Ortega-Sanchez A, Molina-Molina A, Molina R, Rosenfield M, Jiménez R (2022). Blue-blocking filters do not alleviate signs and symptoms of digital eye strain. Clin Exp Optom.

[43] BOE n97, Real Decreto 486/1997, de 14 de abril, por el que se establecen las disposiciones mínimas de seguridad y salud en los lugares de trabajo

