# Supplementary material for: Computer vision syndrome-related symptoms in presbyopic computer workers
Source: Int Ophthalmol. 2023 Apr 27;43(9):3237–45. doi: 10.1007/s10792-023-02724-z (PMC10133911; doi:10.1007/s10792-023-02724-z)
Supplement: Supplementary file 1 — Supplementary file1 (DOCX 35 KB) [file 10792_2023_2724_MOESM1_ESM.docx]

**Appendix 1. Questionnaire**

**I: General information**

1. Age: …………………

2. Gender:

1. Male
2. Female

3. Do you use the computer as your main work tool?

1. Yes
2. No

**II*: General characteristics of computer use**

Participants could continue the questionnaire if they were between 45 and 65 years old and if their main work tool was a computer.

1. Where do you work from most of the time?

1. Office
2. Remote working at home

2. What type of computer do you usually use for work?

1. Desktop computer
2. Laptop computer
3. Both

3. How many hours do you use the computer at work?

1. Less than 6 hours per day
2. 6 or more hours per day

4. How often do you take breaks during your computer work? (A break is defined as a recess of at least 5 minutes).

1. Every 30 minutes
2. Every hour
3. Every more than an hour
4. I do not take breaks

5. How many hours in total do you use electronic devices per day? (Computer, tablet, smartphone, other...)?

1. Between 0 and 5 hours per day
2. Between 5 and 10 hours a day
3. More than 10 hours a day

**III: Data on optical correction**

1. Do you wear any optical correction? (In general, for distance and/or near)

1. Glasses
2. Contact lenses
3. Both
4. I don't wear any because I have had refractive surgery
5. None
6. Other

2. Do you use optical correction all the time?

- 1. Yes
  2. No, only for distance viewing
  3. No, only for near viewing
  4. I have no optical correction

3. What correction do you use for presbyopia? (The correction you usually use for near vision tasks).

- 1. Single near-vision lenses
  2. Bifocal lenses
  3. Progressive lenses
  4. Occupational lenses
  5. None (had refractive surgery)
  6. None at near-vision (myopia at far distance)
  7. Contact lenses

**IV: Visual symptoms associated with computer use**

1. Do you usually notice these eye symptoms? Indicate the severity of each symptom.

|  | None of the time | Some of the time | Half of the time | Most of the time | All of the time |
| --- | --- | --- | --- | --- | --- |
| Blurred vision while viewing the computer |  |  |  |  |  |
| Blurred distance vision after computer work |  |  |  |  |  |
| Difficulty or slowness in refocusing from one distance to another |  |  |  |  |  |
| Irritated or burning eyes |  |  |  |  |  |
| Dry eyes |  |  |  |  |  |
| Eyestrain |  |  |  |  |  |
| Headache |  |  |  |  |  |
| Tired eyes |  |  |  |  |  |
| Sensitivity to bright lights |  |  |  |  |  |
| Eye discomfort |  |  |  |  |  |

2. If you telework from home, have you noticed more or a worsening of visual symptoms compard to when you worked in your office?

1. Yes
2. No
3. I have always teleworked
4. I work from office

**V: Ergonomic conditions for working**

1. In general, do you have adequate lighting in your workspace? (A workspace is considered to be adequately illuminated if it is fully illuminated, homogeneously illuminated and without shadows).

1. Yes
2. No
3. I don't know

2. Do you have an adequate workspace? (A workspace is considered adequate if it is comfortable, ventilated, and spacious).

1. Yes
2. No
3. I don't know

3. What is the position of the screen in relation to your eye level? (The elevation of the eye level in relation to the centre of the screen).

1. At eye level
2. Above the eye level
3. Below the eye level

4. Do you feel neck pain after working at the computer?

1. Yes
2. No
3. I don't know

5. Do you experience back pain after working at the computer?

1. Yes
2. No
3. I don't know
